# Small GTPase FoSec4-Mediated Protein Secretion Is Important for Polarized Growth, Reproduction and Pathogenicity in the Banana Fusarium Wilt Fungus *Fusarium odoratissimum*

**DOI:** 10.3390/jof8080880

**Published:** 2022-08-20

**Authors:** Yuru Zheng, Pingting Guo, Huobing Deng, Yaqi Lin, Guilan Huang, Jie Wu, Songmao Lu, Shuai Yang, Jie Zhou, Wenhui Zheng, Zonghua Wang, Yingzi Yun

**Affiliations:** 1 State Key Laboratory of Ecological Pest Control for Fujian and Taiwan Crops, Ministerial and Provincial Joint Innovation Centre for Safety Production of Cross-Strait Crops, Fujian Agriculture and Forestry University, Fuzhou 350002, China; 2 Fujian Institute for Food and Drug Quality Control, Fuzhou 350001, China; 3 Fuzou Institute of Oceanography, Minjiang University, Fuzhou 350108, China

**Keywords:** *Fusarium odoratissimum*, Rab GTPase, interaction, biofunctions, gene deletion

## Abstract

Apical secretion at hyphal tips is important for the growth and development of filamentous fungi. In this study, we analyzed the role of the Rab GTPases FoSec4 involved in the secretion of the banana wilt fungal pathogen *Fusarium odoratissimum*. We found that the deletion of *FoSEC4* affects the activity of extracellular hydrolases and protein secretion, indicating that FoSec4 plays an important role in the regulation of protein secretion in *F. odoratissimum*. As a typical Rab GTPase, Sec4 participates in the Rab cycle through the conversion between the active GTP-bound state and the inactive GDP-bound state, which is regulated by guanine nucleate exchange factors (GEFs) and GTPase-activating proteins (GAPs). We further found that FoSec2 can interact with dominant-negative FoSec4 (GDP-bound and nucleotide-free form, FoSec4DN), and that FoGyp5 can interact with dominant active FoSec4 (GTP-bound and constitutively active form, FoSec4CA). We evaluated the biofunctions of FoSec4, FoSec2 and FoGyp5, and found that FoSec4 is involved in the regulation of vegetative growth, reproduction, pathogenicity and the environmental stress response of *F. odoratissimum*, and that FocSec2 and FoGyp5 perform biofunctions consistent with FoSec4, indicating that FoSec2 and FoGyp5 may work as the GEF and the GAP, respectively, of FoSec4 in *F. odoratissimum.* We further found that the amino-terminal region and Sec2 domain are essential for the biological functions of FoSec2, while the carboxyl-terminal region and Tre-2/Bub2/Cdc16 (TBC) domain are essential for the biological functions of FoGyp5. In addition, FoSec4 mainly accumulated at the hyphal tips and partially colocalized with Spitzenkörper; however, FoGyp5 accumulated at the periphery of Spitzenkörper, suggesting that FoGyp5 may recognize and inactivate FoSec4 at a specific location in hyphal tips.

## 1. Introduction

Vesicular trafficking is essential for the growth and development of filamentous fungi, and participates in the transport of extracellular signals entering the cell and the secretion of intracellular substances to the outside [1]. Rab GTPases play important roles in different stages of cellular vesicle trafficking [2,3,4]. Sec4/Rab8 was the first Rab GTPase identified in *Saccharomyces cerevisiae* [5,6], and plays a central role in polarized secretion from the Golgi to the plasma membrane by regulating the assembly and docking of the exocyst complex at the target point for secretion during the final stage of exocytosis [7,8]. Studies in filamentous fungi have also found that Sec4 homologous proteins play important roles in regulating the secretion of extracellular proteins in *Aspergillus niger*, *Aspergillus fumigatus*, *Botrytis cinerea*, *Colletotrichum lindemuthianum* and *Magnaporthe oryzae* [9,10,11,12,13,14].

As a typical Rab GTPase, the protein Sec4 participates in the Rab cycle through the conversion between the active GTP-bound state and the inactive GDP-bound state, which is regulated by guanine nucleate exchange factors (GEFs) and GTPase-activating proteins (GAPs) [15,16,17]. GEFs can effectively catalyze the exchange of GDP for GTP to activate Rab GTPases, and GAPs inactivate Rabs by accelerating the hydrolysis of GTP [18,19]. Studies in *S. cerevisiae* have identified some upstream regulators related to the activation and inactivation of Sec4. In *S. cerevisiae*, Sec2 serves as a GEF of Sec4 and is essential for growth and the secretory pathway involving vesicular trafficking from the Golgi to the plasma membrane [20,21]. The Sec2 domain in Sec2 is highly conserved and confers GEF activity to Sec4 [22,23]. Four proteins, including Gyp1, Gyp5, Msb3 and Msb4, have been identified as GAPs of Sec4 in *S. cerevisiae* [24,25,26]. These four proteins all contain the Tre-2/Bub2/Cdc16(TBC) domain, which consists of approximately 200 amino acids and conserved IxxDxxR and YxQ motifs [27].

In filamentous fungi, the biofunctions of these Sec4 homologs have been analyzed by gene deletion and mutant phenotype analyses in several fungal species, including *A. niger*, *A. fumigatus*, *B. cinerea*, *C. lindemuthianum*, *M. oryzae*, *Fusarium graminearum* and *Fusarium verticillioides* [9,10,11,12,13,14,28], and the above studies indicate that Sec4 homologs are important for the vegetative growth and reproduction of filamentous fungi. However, to date, compared with information regarding the biological functions of Sec4 homologs in filamentous fungi, their regulators, including GEFs and GAPs of Sec4 homologs, are not well understood, and a putative GEF and GAP of the Sec4 homologous protein FgRab8 was only recently identified in the wheat head blight fungus *F. graminearum* [29,30]. Huawei Zheng et al. found that FgSec2A (the homolog of *S. cerevisiae* Sec2) acts as a GEF of FgRab8, and that FgMsb3 (the homolog of *S. cerevisiae* Msb3 and Msb4) acts as a GAP of FgRab8 [29,30]. Both FgSec2A and FgMsb3 are required for polarized growth, secondary metabolism and the pathogenicity of *F. graminearum* [29,30]. *AnSEC2*, a homologous gene of *S. cerevisiae SEC2*, was also identified and deleted in *A. niger*; however, the deletion of AnSec2 does not result in a clear phenotype, and the relationship between AnSec2 and AnSec4 has not been identified [31]. In *Neurospora crassa*, only one homolog to *S. cerevisiae*, Msb3 and Msb4, was found, Gyp3, and it plays an important role in maintaining normal fungal cell growth and morphology; however, the relationship between Gyp3 and Rab GTPases has not been determined [32].

*Fusarium oxysporum* f. sp. *cubense* (Foc), the fungal pathogen causing *Fusarium* wilt of banana (Musa spp.), also known as “Panama disease”, is one of the most devastating plant diseases worldwide [33,34]. Foc is a soil-borne pathogen, which can infect the xylem from the banana roots, and induces wilt and kills banana plants. Based on host cultivar specificity, Foc can be classified into four races. Foc race 1 (Foc1) nearly destroyed the global banana trade in the 19th century before the development of the Foc1-resistant banana cultivar Cavendish [34]. Foc race 4 (Foc4) is the most recently identified and can be further subdivided into subtropical race 4 (SR4) and tropical race 4 (TR4). FocTR4 is a serious emerging threat to banana cultivation due to its strong virulence, affecting nearly all banana cultivars, including Cavendish [35]. Recently, Maryani et al. revised the taxonomy of Foc and designated Foc TR4 as *Fusarium odoratissimum* [36]. Studies investigating the pathogenicity of *F. odoratissimum* have found that effectors (such as Six1 and Six8), mycotoxins (such as fusaric acid and beauverin) and a suite of cell wall-degrading and other hydrolytic enzymes are involved in its infection process [37,38]. It is widely agreed that these virulence factors are secreted from the fungal pathogen into the host tissue during pathogenesis, but the detailed regulatory mechanisms underlying secretion are not clearly understood. In a previous study, we explored the roles of the exocyst in the regulation of hydrolytic enzyme secretion at the hyphal tips and septa in *F. odoratissimum* [39]. In this study, we focused on the GTPase FoSec4 in *F. odoratissimum*, and we not only explored the role of FoSec4 in the growth, development and virulence of *F. odoratissimumm*, but also identified two interactors (FoSec2 and FoGyp5) of FoSec4 and analyzed their biological functions in *F. odoratissimum.*

## 2. Materials and Methods

### 2.1. Strains and Culture Conditions

*Fusarium odoratissimum* strain 58, which was isolated, identified, and sequenced in our previous studies [40,41], was used as the wild-type (WT) strain in this study. The genome information of the WT strain was previously deposited into BIG Sub under accession number PRJCA001282 (http://bigd.big.ac.cn/bioproject/browse/PRJCA001282, accessed on 25 April 2019). All strains used in this study (Table S1) were stored as mycelial suspensions in 20% glycerol solution at −80 °C. Complete medium (CM) and minimal medium (MM) were used to test the vegetative growth rates [41]. Liquid potato dextrose broth (PDB) and Spezieller Nährstoffarmer agar (SNA) were used in the conidiation assays [42]. Czapek–Dox liquid medium (3 g of NaNO_3_, 1 g of K_2_HPO_3_, 0.5 g of MgSO_4_, 0.5 g of KCl, 0.01 g of FeSO_4_, 30 g of sucrose and 1 L of water) was used to investigate the production of fusaric acid [43].

### 2.2. Growth and Conidiation Assays

For the growth rate assays on medium plates, fresh mycelial plugs (5 mm in diameter) of each strain obtained from the periphery of a 3-day-old colony were inoculated on CM or MM agar plates. For growth tests under various stress conditions, different CM plates were amended with the following compounds: NaCl, H_2_O_2_, sodium dodecyl sulfate (SDS), Calcofluor White (CFW) and Congo Red (CR) at concentrations indicated in the figure legends. After a 3-day incubation at 28 °C, the colonies of each strain were measured and photographed. Inhibition rate = (diameter of untreated strain − diameter of treated strain)/(diameter of untreated strain) × 100%. For the induction of microcondia, five fresh mycelial plugs were inoculated in a 100 mL flask containing 50 mL of PDB. The flasks were incubated at 28 °C for 3 days in a shaker (180 rpm). For the induction of macrocondia, fresh mycelial plugs of each strain were inoculated on SNA plates at 28 °C for 14 days. For the induction of chlamydospores, five fresh mycelial plugs were inoculated in a 100 mL flask containing 50 mL of SN liquid media. The flasks were incubated at 28 °C for 14 days in a shaker (110 rpm). The filtered and collected mycelia were broken by ultrasound for 10 min, and the chlamydospores were released. The number of spores was determined in each strain using a hemocytometer. The method of using a hemocytometer was briefly described as follows. Firstly, a coverslip was placed over the counting surface of the hemocytometer and the conidia suspension (10 μL) was introduced from the edge of the coverslip; then, the conidia in each of the four outside squares (1 × 1 mm square area) of the hemocytometer was counted. Since 1 cm^3^ is equivalent to 1 mL, the conidia concentration can be determined using the following equation: total number of conidia/mL = average cell count per square × dilution factor × 10^4^. The experiment was repeated three times independently, and statistical analyses were performed according to Duncan’s test.

### 2.3. Construction of Gene Deletion Mutants and Complementation Strains

The double-joint PCR approach was used to generate the gene-replacement constructs for the deletion mutants in *F. odoratissimum* [44]. The primers used to amplify the upstream and downstream fragments of all genes are listed in Table S2. For complementation, we amplified the ORF with its native promoter using the primer pairs in Table S2, cloned it into pKNT vectors using a One Step Cloning Kit (Vazyme Biotech, Nanjing, China), and verified it using a sequence analysis. Each construct was transformed into protoplasts of the target mutant, along with a neomycin resistance gene. The Δ*FoSEC2-1*, Δ*FoSEC2-2*, Δ*FoSEC2-3*, Δ*FoGYP5-1*, Δ*FoGYP5-2* and Δ*FoGYP5-3* strains were constructed by amplifying sequences using the primer pairs listed in Table S2, cloned into pKNT vectors, verified by PCR and DNA sequencing, and then transformed into protoplasts of the target mutant.

### 2.4. Yeast Two-Hybrid (Y2H) Assay

FoSec4WT-BD (wild-type), FoSec4CA-BD (Q69L) and FoSec4DN-BD (N121I) were cloned into the pGBKT7 (bait construct) vector using the primers listed in Table S2. Then, cDNA was cloned into the pGADT7 (prey construct) vector using the primers listed in Table S2, amplified from the cDNA of the *F. odoratissimum* wild-type strain. The Y2H assay was carried out according to the MATCHMAKER GAL4 Two-Hybrid System 3 (Clontech). The pairs of yeast two-hybrid plasmids were cotransformed into the yeast strain AH109 with carrier DNA following a previously reported protocol [45]. pGADT7-T and pGADT7-p53 were used as positive controls, pGADT7-T and pGBKT7-Lam were used as negative controls, pGADT7 and pGBKT7 were used as blank controls, and AD (pGADT7), FoSec4 WT, FoSec4CA and FoSec4DN were used as self-activated controls. The yeast transformants were assayed to determine their growth on SD-Trp-Leu-His-Ade medium.

### 2.5. Virulence Assays

Banana plantlets (Cavendish banana, AAA cultivar) at the five-leaf stage were cultivated in a glasshouse (temperature, 25 °C; light, 12 h; humidity, 75%). For banana root infection, the *F. odoratissimum* strains were cultured in PDB medium for 3 days to induce conidia formation. Before inoculation, the banana plants were pulled out from the soil, then the roots were washed by running water, and the root tips were cut off; the wounded roots were immersed in a conidial suspension (10^6^ conidia/mL) for 8 h, planted in vermiculite, and maintained in a growth chamber. In total, 10 banana plantlets were used in each treatment. After 6 weeks of inoculation, the internal score was used to measure the discolored corm area of individual plants, as previously described by Widinugraheni et al. [37]. For the banana leaf infection assays, the leaf surface was sterilized using 75% ethyl alcohol, the inoculation site (5 mm diameter) on the leaf was pricked for almost 25 pinholes and a fresh mycelial plug (5 mm diameter) was put on it, and then infection symptoms were observed 3–5 days after inoculation. These experiments were repeated three times independently.

### 2.6. Extraction and Collection of Secreted Protein

To induce the secretion of extracellular proteins, modified Czapek–Dox liquid medium, in which sucrose was replaced with apple pectin, was used to culture the *F. odoratissimum* strains. Conidia were collected from the fungal colonies cultured on PDB medium for 3 days, washed twice with ddH_2_O, and inoculated in Czapek–Dox liquid medium at an initial concentration of 10^6^ conidia/mL. After incubation at 28 °C and 160 rpm for 3 days, the mycelium and conidia were removed. Residual impurities were removed by filtering through 0.2 μm syringe filters. The method used for the extraction and collection of secreted protein was previously described [46].

### 2.7. Fusaric Acid Production Assay

The method used for the quantification of the fusaric acid content was previously described [47]. Briefly, five fresh mycelial plugs were inoculated in 100 mL of Czapek–Dox media and incubated at 25 °C in a shaker at 110 rpm for 15 days, and the supernatant was collected by centrifugation. Then, the pH of the supernatant was adjusted to 3.0 with HCl, and the solution was extracted with ethyl acetate three times. The combined ethyl acetate fractions were dried on a rotary evaporator, and the resultant residues were redissolved in 1 mL of methanol and filtered through 0.45 µm filters. A Waters 2695 RP-HPLC system was employed to analyze fusaric acid, and elution was carried out using a mobile phase comprising 65% methanol and 35% double-distilled water with 30 mM H_3_PO_4_ (pH 7.34) for 20 min (flow rate of 1 mL/min) with a UV detector at 270 nm.

### 2.8. Extracellular Hydrolase Activity Detection

The method used for the detection of extracellular hydrolase activity has been previously described [47]. Briefly, three fresh mycelial plugs were inoculated in 100 mL of Czapek–Dox medium, in which sucrose was replaced with 1% bran, for approximately 7 days at 25 °C in a shaker at 110 rpm, and the culture filtrates were used for the measurement of extracellular enzyme activities. The activities of amylase, polygalacturonase (PG) and endonuclease (EG) were determined by using the 3,5-dinitrosalicylic acid (DNS) method, as previously described [48]. The activity of laccase was determined by the ABTS method [49]. The dried weights of the harvested mycelia were measured to normalize the enzyme activities.

### 2.9. Light and Epifluorescence Microscopy

Fresh mycelia were collected for the live cell imaging by shaking mycelia for 24 h in CM. A Nikon A1R laser scanning confocal microscope system (Nikon, Japan) was used for the live-cell imaging. GFP excitation was performed with 488 nm light (Em. 525/40 nm). The hyphal tips were visualized by staining with FM4-64 at a final concentration of 10 mg/mL with 405 nm light (Em. 452/45 nm) and photographed.

## 3. Results

### 3.1. FoSec2 and FoGyp5 Interact with FoSec4DN and FoSec4CA, Respectively

Using the amino acid sequence of *S. cerevisiae* Sec4 as the target sequence, we identified the homologous protein FoSec4 (FOIG_08151) in *F. odoratissimum* by protein blast (https://blast.ncbi.nlm.nih.gov/Blast.cgi accessed on 25 April 2019) in the NCBI database. Homologs of Sec4 in other filamentous fungi (including the seven filamentous fungi *A. niger*, *A. fumigatus*, *B. cinereal*, *C. lindemuthianum*, *M. oryzae*, *F. graminearum* and *F. verticillioides,* and four yeasts, *Schizosaccharomyces pombe*, *Yarrowia lipolytica*, *Candida albicans* and *Parastagonospora noaorum*) were also identified. The multiple sequence alignment indicated that the above proteins all possess the signature motifs of Rab GTPases (Figure S1), and clearly, compared with the five yeast species, the Sec4 homologs from the eight filamentous fungi share more conserved amino acid sequences (Figure S2A).

**Figure 1:**
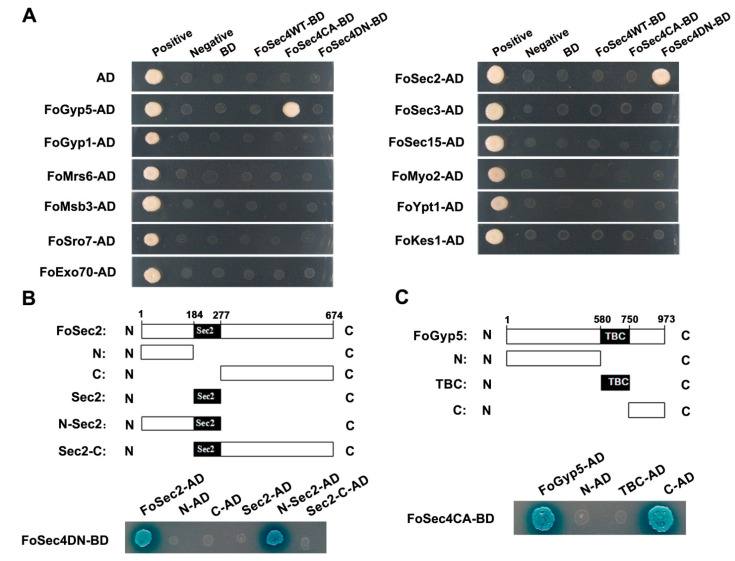
FoSec2 and FoGyp5 interact with FoSec4DN and FoSec4CA, respectively. (**A**) Yeast transformants expressing 12 proteins with FoSec4WT-BD, FoSec4CA-BD(Q69L) and FoSec4DN-BD(N123I) were grown on SD-Leu-Trp-His-Ade plates. FoSec2 specifically interacts with FoSec4DN, and FoGyp5 specifically interacts with FoSec4CA. (**B**) Schematic drawing of full-length FoSec2, the N-terminus, the C-terminus, the Sec2 domain, N-Sec2 and Sec2-C. The N-Sec2 region (amino acids 1–277) is essential for the interaction between FoSec2 and FoSec4DN. (**C**) Schematic drawing of FoGyp5-full length, the N-terminus, the C-terminus and the TBC domain. The C-terminus (amino acids 750–973) is essential for the interaction between FoGyp5 and FoSec4CA.

To determine whether the action network of *S. cerevisiae* Sec4 is also present in *F. odoratissimum*, homologs of 12 interactors of *S. cerevisiae* Sec4 were identified in *F. odoratissimum* (Table 1); then, we demonstrated their interaction with FoSec4 using a yeast two-hybrid (Y2H) assay (Figure 1A). From the results of the Y2H assays, we found that among the 12 proteins, only FoSec2 can interact with dominant negative FoSec4 (GDP-bound and nucleotide-free form, FoSec4DN), and that only FoGyp5 can interact with dominant active FoSec4 (GTP-bound and constitutively active form, FoSec4CA), while both FoSec4 and FoGyp5 cannot interact with native FoSec4 (Figure 1A). The above results suggest that FoSec2 and FoGyp5 may work as the GEF and the GAP, respectively, of FoSec4 in *F. odoratissimum*.

FoSec2 contains a Sec2 domain, and FoGyp5 possesses a typical TBC domain (Figure 1B,C). To investigate whether these domains are involved in the interactions with FoSec4, we generated five FoSec2 variants and three FoGyp5 variants (Figure 1B,C). Different combinations of these FoSec2 and FoGyp5 constructs were transformed into *S. cerevisiae* AH109 with three forms of FoSec4 (native, dominant active or negative) for the Y2H assays. Based on the Y2H results, we found that the amino-terminal portion (amino acids 1–277) of FoSec2, including the amino-terminus and the Sec2 domain, is essential for fostering the interaction between FoSec2 and FoSec4DN, and that the carboxyl-terminal portion (amino acids 750–973) of FoGyp5 without the TBC domain is required for fostering the interaction between FoGyp5 and FoSec4CA (Figure 1B,C).

### 3.2. FoSec4 and FoGyp5 Are Well Distributed in the Cytoplasm but Exhibit Different Patterns in the Hyphal Tips of F. odoratissimum

**Figure 2:**
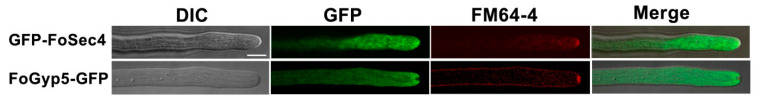
Subcellular localization of GFP-FoSec4 and FoGyp5-GFP in the hyphae of *F. odoratissimum*. Shown are confocal fluorescence images indicating that the two proteins are located and well distributed in the cytoplasm but exhibit different patterns in the hyphal tips. GFP-FoSec4 colocalized with Spitzenkörper, but FoGyp5-GFP accumulated at the periphery of Spitzenkörper. Bars = 10 μm.

To further analyze the biofunctions of FoSec4, FoSec2 and FoGyp5 in *F. odoratissimum*, the genes encoding the above three proteins were deleted. Through a homologous recombination strategy, gene deletion mutants for *FoSEC4*, *FoSEC2* and *FoGYP5* were generated and confirmed by Southern blot assays (Figure S3). Since an interaction exists between FoSec2 and FoSec4DN and between FoGyp5 and FoSec4CA, we wondered about the localization pattern of the three proteins. Therefore, FoSec4-GFP, FoSec2-GFP and FoGyp5-GFP fusion constructs were generated and transformed into the corresponding gene deletion mutants. At first, FoSec4, FoSec2 and FoGyp5 were all labeled with GFP at the C-terminus, and we succeeded to observe the cellular localization of FoGyp5 in the transformants with FoGyp5-GFP; however, we failed to find the GFP signals in the transformants with FoSec4-GFP or FoSec2-GFP. Then, we labeled the GFP on the N-terminus of FoSec4 and FoSec2, and observed the cellular localization of FoSec4 in the transformants with GFP-FoSec4; however, we still could not find the GFP signals in the transformants with GFP-FoSec2. All generated gene-complemented strains (Δ*FoSEC4-C*, Δ*FoSEC2-C* and Δ*FoGYP5-C*) displayed a wild-type morphology in the phenotype assays. Then, the localization of GFP-FoSec4 and FoGyp5-GFP in growing *F. odoratissimum* hyphae was monitored, and both were located and well distributed in the cytoplasm, but exhibited different patterns in the hyphal tips (Figure 2). In filamentous fungi, the polarized traffic of secretory vesicles to the vesicle supply center (VSC) occurs at the end of polarized secretion, and the Spitzenkörper, a vesicle-dense region in the hyphal tip, acts as a VSC and can be stained with the dye FM4-64 as a bright spot at the center of the apical dome [50,51]. Thus, we further observed the cellular localization of FoSec4-GFP and FoGyp5-GFP at the hyphal tips by dying with FM4-64. As shown in Figure 2, GFP-FoSec4 mainly accumulated at the tip of the mycelium and partially colocalized with Spitzenkörper, but a FoGyp5-GFP signal was not observed in the Spitzenkörper area. Additionally, FoGyp5-GFP accumulated at the periphery of Spitzenkörper. The above results suggest that FoGyp5 may recognize and inactivate FoSec4 at a specific location in hyphal tips.

### 3.3. FoSec4 and FoSec2 Are Important for Vegetative Growth and Polarized Growth in F. odoratissimum

**Figure 3:**
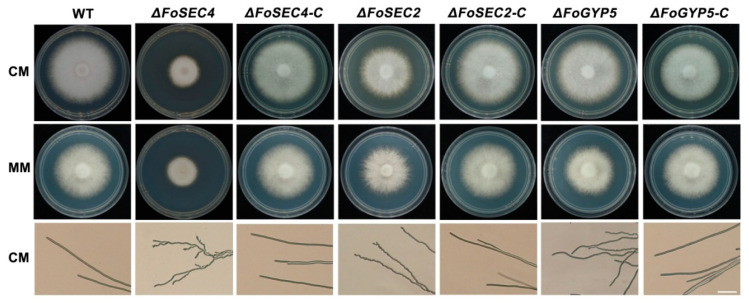
Vegetative growth of the wild-type (WT), Δ*FoSEC4*, Δ*FoSEC2*, Δ*FoGYP5*, Δ*FoSEC4-C*, Δ*FoSEC2-C* and Δ*FoGYP5-C* strains. All strains were cultured on CM and MM plates at 28 °C for 3 days. The hyphae on the CM plate were photographed by microscopy. Bar = 50 µm.

To evaluate the roles of FoSec4, FoSec2 and FoGyp5 in the vegetative growth of *F. odoratissimum*, the wild-type, gene deletion mutants and complemented strains were incubated on complete medium (CM) and minimal medium (MM) solid agar plates for 3 days. The Δ*FoSEC4* and Δ*FoSEC2* colonies exhibited a decrease in the colony diameters of approximately 40% and 18%, respectively, compared with those of the wild-type and complemented strains, while only Δ*FoGYP5* showed a slightly smaller colony than the wild-type and complemented strains on both CM and MM plates (Figure 3). We further observed the hyphal morphology of each strain, and found that the hyphae of Δ*FoSEC4* and Δ*FoSEC2* became curved compared with those of the wild-type and complemented strains (Figure 3A). The above results show that FoSec4 and FoSec2 are required for vegetative growth and polarized growth in *F. odoratissimum*.

### 3.4. FoSec2 and FoSec4 Participate in the Regulation of the Reproduction Process of F. odoratissimum

**Figure 4:**
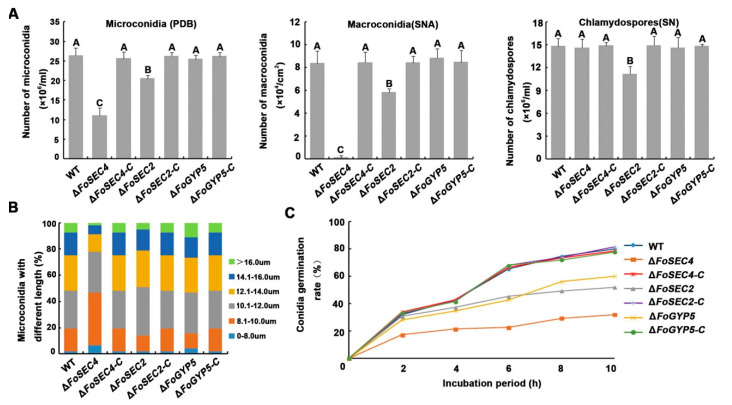
FoSec2 and FoSec4 participate in the regulation of the reproduction process of *F. odoratissimum.* (**A**) Microconidia were measured by counting the number in PDB culture after 3 days. Macroconidia were measured by counting the number in SNA plate culture after 14 days. Chlamydospores were measured by counting the number in SN after 14 days. The error bars represent the standard deviation (SD) of three replicates, and the values on the bars followed by the same letter are not significantly different at *p* = 0.01. (**B**) The length of microconidia in all the above strains. (**C**) The germination rates of microconidia of each strain after incubation for 2 h, 4 h, 6 h, 8 h and 10 h.

A sexual reproduction stage has not been found in the life process of *F. odoratissimum*; thus, asexual spores play a key role in the fungal disease life cycle, which includes three types of spores—microconidia, macroconidia and chlamydospores [34]. Therefore, to assess the reproduction abilities of Δ*FoSEC4*, Δ*FoSEC2* and Δ*FoGYP5* compared to that of the wild-type strain, these three strains were cultured under three different conditions, including in PDB liquid media for 3 days for the induction of microconidia, SNA plates for 14 days for the induction of macroconidia, and SN liquid media for 14 days for the induction of chlamydospores. As shown in Figure 4, microconidia and macroconidia production in Δ*FoSEC4* and Δ*FoSEC2* was significantly reduced compared with that in the wild-type and complemented strains; especially Δ*FoSEC4*, which produced almost no macroconidia. However, the deletion of *FoSEC4* did not affect chlamydospore production, while the chlamydospore number of Δ*FoSEC2* was reduced. In addition, the deletion of Δ*FoGYP5* did not affect the production of the three types of spores in *F. odoratissimum.* The above results imply that FoSec2 and FoSec4 participate in the regulation of the reproduction process of *F. odoratissimum.* We further observed the morphology of the microconidia, macroconidia and chlamydospores of each strain, and found that deletion of the target gene does not affect the spores’ morphology (Figure S4), except that the microconidia from Δ*FoSEC4* displayed a slightly smaller size than the wild-type and complemented strains (Figure 4B). In addition, the germination rates of microconidia from the three gene deletion mutants, especially Δ*FoSEC4*, were down regulated compared with those of the wild-type and complemented strains (Figure 4C), implying that these three genes, especially *FoSEC4*, participate in the regulation of the conidial germination process in *F. odoratissimum.*

### 3.5. Deletion of FoSEC4, but Not FoSEC2 or FoGYP5, Leads to Notably Decreased Virulence

**Figure 5:**
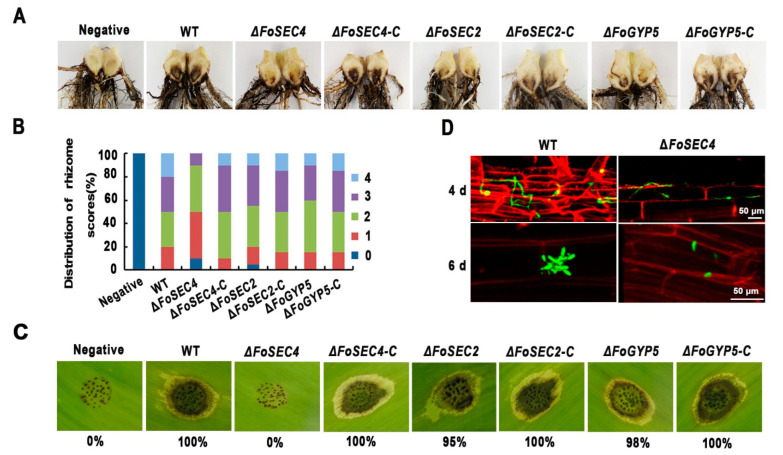
Pathogenicity of the wild-type (WT), Δ*FoSEC4*, Δ*FoSEC2*, Δ*FoGYP5*, Δ*FoSEC4-C*, Δ*FoSEC2-C* and Δ*FoGYP5-C* strains. (**A**) Disease symptoms on the corms of banana tissue plantlets were recorded 2 months after inoculation with the indicated strains and negative control water. (**B**) The percentage of plants in each disease index was scored for corm symptoms as follows: 0 represents no browning in the corm, 1 represents browning in 1% to 25% of the area in the corm, 2 represents browning in 25% to 50% of the area in the corm, 3 represents browning in 50% to 75% of the area in the corm, and 4 represents browning in 75% to 100% of the area in the corm. (**C**) Necrosis spots on banana leaves were recorded 5 days after inoculation with the indicated strains and negative control water. The bottom numbers indicate the relative size of necrotic tissue caused by the indicated strains compared with the WT strain. (**D**) Confocal images of the WT-GFP and Δ*FoSEC4*-GFP strains growing inside banana roots 4 and 6 days after inoculation. Bar = 50 μm.

To evaluate the virulence of Δ*FoSEC4*, Δ*FoSEC2* and Δ*FoGYP5*, all strains were inoculated into the roots of banana plants. Six weeks postinoculation, we observed noticeable vascular discoloration in the corms of the banana plantlets inoculated with conidia of the wild-type and complemented strains. Moreover, the banana plantlets inoculated with Δ*FoSEC4* showed less discoloration in the corm (Figure 5A), while the banana plants inoculated with Δ*FoSEC2* or Δ*FoGYP5* showed only slightly reduced necrosis symptoms in the xylem system (Figure 5A); the disease index analysis confirmed these observations (Figure 5B). We also observed the virulence of all the above strains on banana leaves and found that inoculation of the strains, except for Δ*FoSEC4,* showed clear necrosis spots on the banana leaves; however, the leaf inoculated with Δ*FoSEC4* showed a phenotype similar to that of the negative control (Figure 5C), suggesting that compared with FoSec2 and FoGyp5, FoSec4 plays a more important role in the pathogenicity of *F. odoratissimum*. Therefore, we further observed the infection process of Δ*FoSEC4* by fluorescence microscopy (Figure 5D). The wild-type strain and Δ*FoSEC4* were labeled with GFP and inoculated into the roots of banana plants. After 6 days of inoculation, accumulated infection hyphae and conidia were observed in the roots inoculated with the wild-type strain, but only a few infection hyphae and conidia were found in the roots inoculated with Δ*FoSEC4* (Figure 5D), indicating that Δ*FoSEC4* shows a reduced colonization ability in banana roots.

Fusaric acid (FA), a broad-spectrum phytotoxic compound, is known to contribute to the severity of *Fusarium* diseases, e.g., vascular wilt, damping-off, and root rot, in numerous crops, including bananas [52]. To determine whether FoSec4 is involved in the regulation of FA biosynthesis, we analyzed fusaric acid production by the wild-type strain, Δ*FoSEC4*, and the complemented strains in Czapek–Dox broth via high-performance liquid chromatography (HPLC). However, the deletion of *FoSEC4* did not affect FA biosynthesis (Figure S5), suggesting that FoSec4 is not important for the FA biosynthesis pathway in *F. odoratissimum.*

### 3.6. Deletion of FoSEC4 Results in Attenuated Activities of Extracellular Cell Wall-Degrading Enzymes and Reduced Protein Secretion

**Figure 6:**
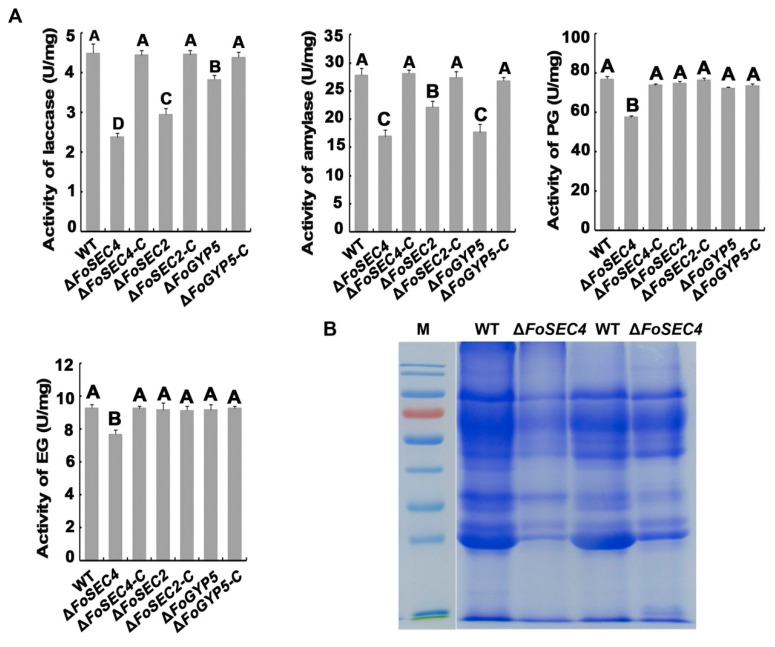
FoSec4, FoSec2 and FoGyp5 are required for protein secretion in *F. odoratissimum*. (**A**) The activities of laccase, amylase, polygalacturonase (PG) and endoglucanase (EG) secreted from the indicated strains after 7 days of cultivation in modified Czapek–Dox medium were detected using the DNS method. The error bars represent the standard deviation (SD) of three replicates, and the values on the bars followed by the same letter are not significantly different at *p* = 0.01. (**B**) Coomassie brilliant blue gel of secreted proteins from the wild-type (WT) and Δ*FoSEC4* strains cultured for 3 days in modified Czapek–Dox liquid medium, in which sucrose was replaced with apple pectin.

Secreted cell wall-degrading enzymes (CWDEs), e.g., cellulase, xylanase, pectinase, and amylase, are important for overcoming physical obstacles, such as the plant cell wall, in the infection process of *F. odoratissimum*. To explore whether the loss of FoSec4 affects the secretion of CWDEs, we evaluated the activity of laccase, amylase, polygalacturonase (PG), and endoglucanase (EG). The deletion of *FoSEC4* resulted in decreased activities of laccase, amylase, PG and EG, while the loss of *FoSEC2* or *FoGYP5* affected only the activities of laccase and amylase (Figure 6A), suggesting that FoSec4 plays a more important role in regulating the secretion of CWDEs. To further confirm the role of FoSec4 in exocytosis, secreted proteins from the wild-type and Δ*FoSEC4* strains were collected. The wild-type and Δ*FoSEC4* strains were incubated in modified Czapek–Dox liquid medium (in which sucrose was replaced with apple pectin) for 3 days, and then the filtrated supernatant was centrifuged to remove the mycelium and conidia. The filtrated mycelium was collected and dried to determine its mass. Since Δ*FoSEC4* grows more slowly than the wild-type strains, the amount of supernatant used for protein extraction was adjusted based on the dry mass obtained from the cultures. The total secreted proteins from the wild-type and Δ*FoSEC4* strains were analyzed by SDS–PAGE. As shown in Figure 6B, the intensity of the protein bands of Δ*FoSEC4* was notably decreased compared with that of the wild-type strain, suggesting that Δ*FoSEC4* secreted fewer extracellular proteins than the wild-type strain.

### 3.7. FoSec4, FoSec2 and FoGyp5 Participate in the Response to Environmental Stressors

**Figure 7:**
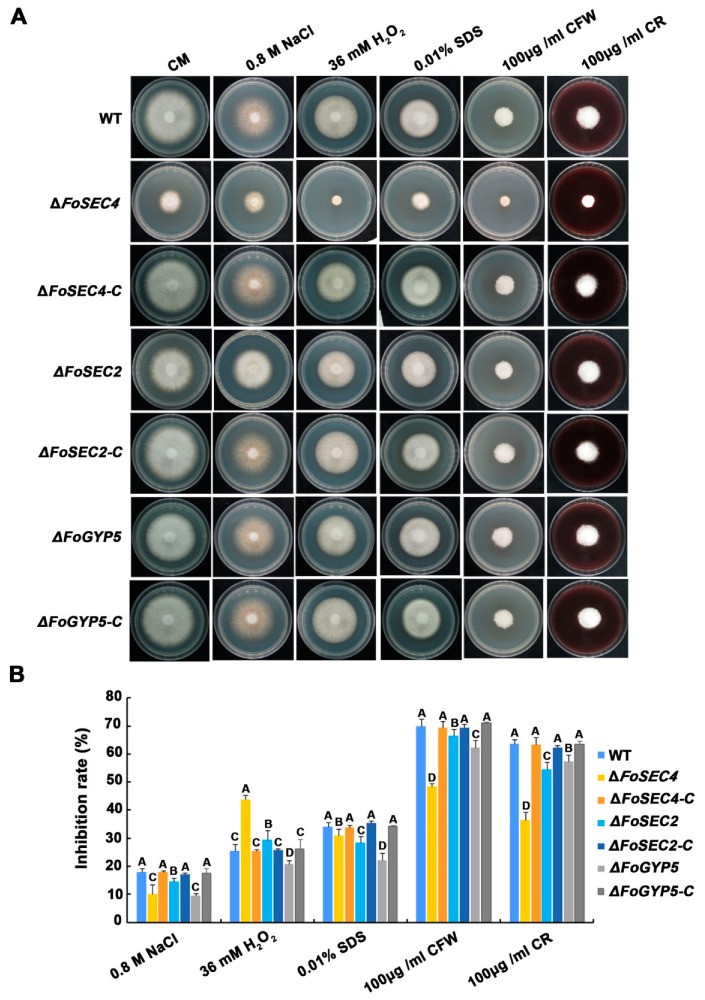
Responses of the wild-type (WT), Δ*FoSEC4*, Δ*FoSEC2*, Δ*FoGYP5*, Δ*FoSEC4-C*, Δ*FoSEC2-C* and Δ*FoGYP5-C* strains to various stress conditions. (**A**) Strains were grown on CM without or with various stress inducers as indicated at 28 °C for 3 days and then photographed. (**B**) Colony diameters were subjected to a statistical analysis. The growth inhibition rate is relative to the growth rate of each untreated control. The error bars represent the standard deviation (SD) of three replicates, and the values on the bars followed by the same letter are not significantly different at *p* = 0.01.

Apical secretion is essential for fungal apical growth, which provides the necessary materials and enzymatic platforms needed for tip cell wall expansion and growth [53]. To determine whether FoSec4, FoSec2 and FoGyp5 are involved in the response to stresses reported to be associated with cell wall integrity, we investigated the growth of all mutants on media containing various pathogenesis-associated stress-mimicking agents, including NaCl, H_2_O_2_, SDS, CFW and CR. Among them, NaCl is an osmotic stress agent, H_2_O_2_ causes oxidative stress, and SDS, CFW and CR impair the cell wall integrity and are detergents used to test the cell wall integrity [54,55,56,57]. The growth of all mutants on media containing NaCl, H_2_O_2_, SDS, CFW and CR was severely inhibited, especially on the media with CFW and CR (Figure 7). All mutants were less sensitive to NaCl, SDS, CFW and CR than the wild-type, implying that FoSec4, FoSec2 and FoGyp5 might negatively influence the response to osmotic stress and the cell wall integrity. Interestingly, we found that the ∆*FoSec4* mutant was more sensitive to H_2_O_2_, while the ∆*FoSec2* and ∆*FoGyp5* mutants were less sensitive to H_2_O_2_, implying that FoSec4, FoSec2 and FoGyp5 are required for the oxidative stress response.

### 3.8. The Amino-Terminal Region and Sec2 Domain Are Essential for the Biological Functions of FoSec2, While the Carboxyl-Terminal Region and TBC Domain Are Essential for the Biological Functions of FoGyp5

**Figure 8:**
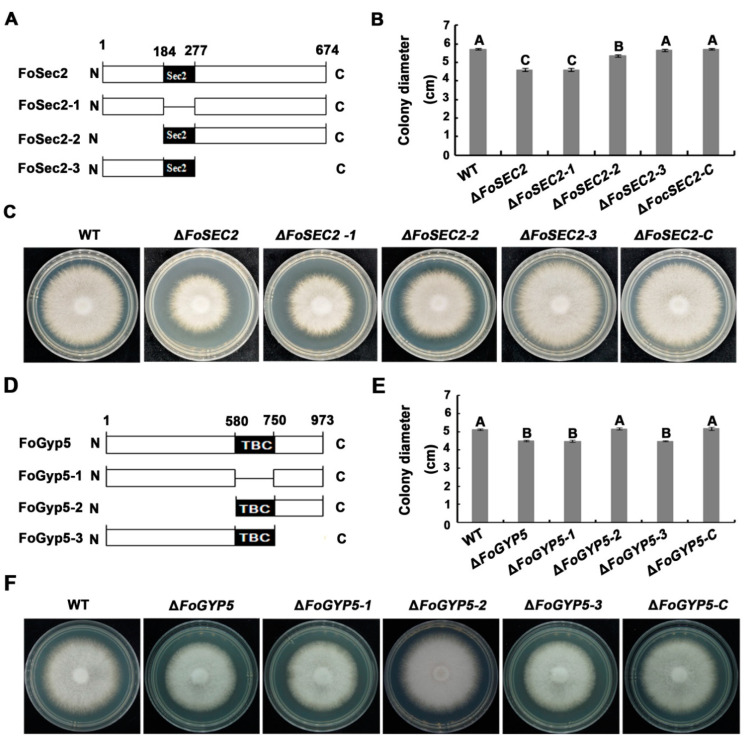
The Sec2 domain and N-termini of FoSec2 were crucial for vegetative growth, and the TBC domain and the C-termini of FoGyp5 were crucial for vegetative growth in *F. odoratissimum*. (**A**) Schematic drawing of the deletion of the Sec2 domain and the N- and C-termini of FoSec2. (**B**) The colony diameters of all FoSec2 domain deletion mutants. The error bars represent the standard deviation (SD) of three replicates, and the values on the bars followed by the same letter are not significantly different at *p* = 0.01. (**C**) Colony morphology of all FoSec2 domain deletion mutants on CM for approximately 3 days. (**D**) Schematic drawing of the deletion of the TBC domain and the N- and C-termini of FoGyp5. (**E**) The colony diameters of all FoGyp5 domain deletion mutants. The error bars represent the standard deviation (SD) of three replicates, and the values on the bars followed by the same letter are not significantly different at *p* = 0.01. (**F**) Colony morphology of all FoGyp5 domain deletion mutants on CM for approximately 3 days.

Based on the earlier results, the amino-terminal region, including the Sec2 domain, and the carboxyl-terminal domain without the TBC domain are essential for the interaction between FoSec2 and FoSec4ND and the interaction between FoGyp5 and FoSec4CA, respectively. We wondered which domain contributes to the biofunctions of FoSec2 or FoGyp5 in *F. odoratissimum*; thus, we generated a series of *FoSEC2* or *FoGYP5* deletion constructs (Figure 8). The truncated *FoSEC2s* or *FoGYP5s* were then transformed into Δ*FoSEC2* or Δ*FoGYP5* for complementation assays. As shown in Figure 8, Δ*FoSEC2-3* (complemented with amino acids 1-277 of FoSec2) recovered the mycelial growth defects of Δ*FoSEC2*, while Δ*FoSEC2-1* (complemented with FoSec2 without the Sec2 domain) and Δ*FoSEC2-2* (complemented with amino acids 184-677 of FoSec2) did not recover the vegetative growth defects of Δ*FoSEC2*, suggesting that the amino-terminal region and Sec2 domain are essential for the biological functions of FoSec2, which is consistent with the regions required for fostering the interaction between FoSec2 and FoSec4DN. Compared with the wild-type strain and Δ*FoGYP5*, Δ*FoGYP5-2* (complemented with amino acids 580–973 of FoGyp5) recovered the mycelial growth defects of Δ*FoGYP5*, but Δ*FoGYP5-1* (complemented with FoGyp5 without the TBC domain) and Δ*FoGYP5-3* (complemented with amino acids 184-677 of FoGyp5) did not recover the vegetative growth defects of Δ*FoGYP5* (Figure 8), implying that both the carboxyl-terminal region and TBC domain are essential for the biological functions of FoGyp5.

## 4. Discussion

Vesicle transport is essential for the secretion of virulence-related factors from *F. odoratissimum* into the banana host; however, the molecular mechanism underlying this process is not well understood. In this study, we identified the Rab GTPase FoSec4, which is important for the protein secretion of *F. odoratissimum*. The deletion of *FoSEC4* resulted in defects in the vegetative growth, reproduction and pathogenicity of *F. odoratissimum*. We further identified two interactors of FoSec4 as follows: FoSec2 interacts with dominant negative FoSec4DN (GDP-bound and nucleotide-free form), and FoGyp5 interacts with dominant active FoSec4CA (GTP-bound and constitutively active form). These two interactors show biofunctions consistent with FoSec4 to regulate vegetative growth and reproduction in *F. odoratissimum,* but display different roles compared with FoSec4 in the regulation of the pathogenicity process of *F. odoratissimum.*

Research investigating Sec4 homologs in filamentous fungi found that Sec4 homologs participate in the regulation of fungal vegetative growth and reproduction [11,14,58]. In our study, we found that the gene deletion mutant of *FoSEC4* shows a notably decreased growth rate and reduced conidia production (Figure 3 and Figure 4). Similar phenotypes were also observed in the gene deletion mutants of *SEC4* homologs from other plant fungal pathogens, including *F. verticillioides*, *F. graminearum*, *B. cinerea* and *M. oryzae* [11,14,28,58]. However, among these fungi, only the loss of *FoSEC4* in *F. odoratissimum* or *FgRAB8* (*SEC4* homolog) in *F. graminearum* led to a reduced conidial germination rate [29]. In addition, the deletion of the *SEC4* homologs in the above plant pathogens led to reduced virulence in the host plants. For the rice blast fungus, the appressorium is essential for *M. oryzae* to penetrate the rice leaf and invade the host plant. The loss of MoSec4 results in decreased turgor pressure in the appressorium, and the biotrophic invasive hyphae in the host cells are more bulbous and compressed in the *MoSEC4* mutant [14]. However, during the infection process of *F. odoratissimum*, the infection hypha from the germinated spores penetrate the surface of the banana roots from the intercellular space of the epidermis and wounds without the production of special structures [59]. We found that the loss of *FoSEC4* does not affect the phenotypes of infection hyphae inside the host cells, but leads to diminished colonization of *F. odoratissimum* (Figure 5D). Mycotoxins are important virulence factors in the pathogenicity of *Fusarium* spp., and the loss of *FgRAB8* or *FvSEC4* affected the production of mycotoxin deoxynivalenol (DON) or fumonisin B1 (FB1) in *F. graminearum* or *F. verticillioides* [28,58]; however, the deletion of *FoSEC4* did not affect the production of fusaric acid in *F. odoratissimum*. The above results suggest that although Sec4 homologs in plant pathogens share conserved biofunctions in the regulation of fungal growth and reproduction, different Sec4 homologs display species-specific diversity involved in pathogenicity.

To further explore the molecular mechanism of FoSec4, we identified the homologs of the *S. cerevisiae* Sec4 interactors in *F. odoratissimum* and demonstrated whether the interaction between the interactors and Sec4 is also present in *F. odoratissimum*. Surprisingly, only two proteins among twelve Sec4 interactor homologs had an interaction with FoSec4, including FoSec2, which interacted with FoSec4DN, and FoGyp5, which interacted with FoSec4CA (Figure 1). Sec2 is a classic GEF of Sec4 in *S. cerevisiae* and binds the switch regions of Sec4 to stimulate nucleotide exchange in Sec4 [20,60]. In *F. graminearum*, FgSec2A (a Sec2 homolog) also interacts with FgRab8DN and acts as the GEF of FgRab8 [29]. FoSec2 shares 27.92% protein identity with Sec2 from *S. cerevisiae*, but shares 89.64% protein identity with FgSec2. The protein structures of FoSec2 and FgSec2A are highly conserved (Figure S2B), and both the amino terminal and Sec2 domains of FoSec2 and FgSec2A are important for protein biofunctions and interactions with Sec4 homologs in *F. odoratissimum* and *F. graminearum*. Sec2 is encoded by an essential gene in *S. cerevisiae*, but both *FoSEC2* and *FgSEC2A* can be deleted in the progenitor strain, and the mutants showed slightly decreased vegetative growth, reproduction and virulence, suggesting that the biofunctions of Sec2 homologs are not conserved between *Fusarium* fungi and yeast species.

In *S. cerevisiae*, the GAP activity of Gyp5 of Sec4 is required for efficient exocytosis, and the loss of Gyp5 results in cold-sensitive slow growth, the accumulation of ER membranes and autophagic processes [61]. In this study, we found that FoGyp5 can interact with FoSec4CA and that the *FoGYP5* gene deletion mutant shows defects in hyphal morphology, stress responses and the secretion of extracellular hydrolases, which is consistent with the corresponding phenotypes of Δ*FoSEC4*. Therefore, we infer that FoGyp5 acts as the GAP of FoSec4, and to the best of our knowledge, this is the first report of an interaction between the Gyp5 homolog and Sec4 homolog in filamentous fungi. In *F. graminearum*, FgMsb3, the homolog of Msb3 in *S. cerevisiae*, works as the GAP of FgRab8 [30], but we did not demonstrate the interaction between the Msb3 homolog and FoSec4 in *F. odoratissimum* (Figure 1). FgMsb3 was specifically located at the hyphal apical dome (HAD) and partially colocalized with Spitzenkörper [30], while FoGyp5 accumulated at the hyphal tips but avoided the Spitzenkörper region, suggesting that different GAPs participate in the inactivation of Sec4 proteins in the hyphal tip area.

Overall, our work and other publications indicate that the working mechanism of Sec4 homologs in filamentous fungi is not conserved between yeast fungi and filamentous fungi. The reasons for the above conclusion may include the observations that Sec4 homologs are mainly involved in apical secretion at the hyphal tips in filamentous fungi, which distinguishes them from yeast fungi. Therefore, further identification of the interactors and regulators of Sec4 homologs in filamentous fungi is important and will advance our understanding of the apical secretion process at the growth site during the development and pathogenicity of filamentous fungi.

## Figures and Tables

**Table 1:** Homologs of 12 interactors of *S. cerevisiae* Sec4 in *F. odoratissimum*.

Protein in *Saccharomyces cerevisiae*	Homologs in *Fusarium odoratissimum*	Protein Identity
Gyp5	FoGyp5 (FOIG_01503)	40.67%
Gyp1	FoGyp1 (FOIG_07346)	39.32%
Mrs6	FoMrs6 (FOIG_15902)	26.32%
Msb3	FoMsb3 (FOIG_04024)	41.76%
Sro7	FoSro7 (FOIG_01252)	28.13%
Exo70	FoExo70 (FOIG_06688)	23.43%
Sec2	FoSec2 (FOIG_11948)	27.92%
Sec3	FoSec3 (FOIG_06126)	36.14%
Sec15	FoSec15 (FOIG_08908)	24.20%
Myo2	FoMyo2 (FOIG_02660)	46.22%
Ypt1	FoYpt1 (FOIG_07693)	79.01%
Osh4	FoKes1 (FOIG_10637)	45.56%

## Data Availability

Not applicable.

## Supplementary Materials

The following are available online at https://www.mdpi.com/article/10.3390/jof8080880/s1, Figure S1: Multiple alignment of Sec4 homologs in some fungi species; Figure S2: Phylogenetic analysis of Sec4, Sec2 and Gyp5 homologs in some fungi species; Figure S3: Southern blot analyses of targeted gene deletion mutants; Figure S4: The morphology of the microconidia, macroconidia and chlamydospores in the wild-type (WT), Δ*FoSEC4*, Δ*FoSEC2*, Δ*FoGYP5*, Δ*FoSEC4-C*, Δ*FoSEC2-C* and Δ*FoGYP5-C* strains; Figure S5: Fusaric acid production levels by wild-type (WT), Δ*FoSEC4* and Δ*FoSEC4-C* strains were analyzed by HPLC. Fusaric acid levels (mg g^−1^) were measured per mycelial dry weight; Table S1: The strains used in this study; Table S2: Primers used in this study.

## References

[1] Stenmark H. (2009). Rab GTPases as coordinators of vesicle traffic. Nat. Rev. Mol. Cell Biol..

[2] Grosshans B.L., Ortiz D., Novick P. (2006). Rabs and their effectors: Achieving specificity in membrane traffic. Proc. Natl. Acad. Sci. USA.

[3] Schwartz S.L., Cao C., Pylypenko O., Rak A., Wandinger-Ness A. (2007). Rab GTPases at a glance. J. Cell Sci..

[4] Fukuda M. (2008). Regulation of secretory vesicle traffic by Rab small GTPases. Cell. Mol. Life Sci. CMLS.

[5] Novick P., Field C., Schekman R. (1980). Identification of 23 complementation groups required for post-translational events in the yeast secretory pathway. Cell.

[6] Gallwitz D., Donath C., Sander C. (1983). A yeast gene encoding a protein homologous to the human c-has/bas proto-oncogene product. Nature.

[7] TerBush D.R., Maurice T., Roth D., Novick P. (1996). The Exocyst is a multiprotein complex required for exocytosis in *Saccharomyces cerevisiae*. EMBO J..

[8] Hsu S.C., Ting A.E., Hazuka C.D., Davanger S., Kenny J.W., Kee Y., Scheller R.H. (1996). The mammalian brain rsec6/8 complex. Neuron.

[9] Punt P.J., Seiboth B., Weenink X.O., van Zeijl C., Lenders M., Konetschny C., Ram A.F., Montijn R., Kubicek C.P., van den Hondel C.A. (2001). Identification and characterization of a family of secretion-related small GTPase-encoding genes from the filamentous fungus *Aspergillus niger*: A putative SEC4 homologue is not essential for growth. Mol. Microbiol..

[10] Powers-Fletcher M.V., Feng X., Krishnan K., Askew D.S. (2013). Deletion of the sec4 homolog srgA from *Aspergillus fumigatus* is associated with an impaired stress response, attenuated virulence and phenotypic heterogeneity. PLoS ONE.

[11] Zhang Z., Qin G., Li B., Tian S. (2014). Knocking out Bcsas1 in *Botrytis cinerea* impacts growth, development, and secretion of extracellular proteins, which decreases virulence. Mol. Plant-Microbe Interact. MPMI.

[12] Siriputthaiwan P., Jauneau A., Herbert C., Garcin D., Dumas B. (2005). Functional analysis of *CLPT1*, a Rab/GTPase required for protein secretion and pathogenesis in the plant fungal pathogen *Colletotrichum lindemuthianum*. J. Cell Sci..

[13] Dumas B., Borel C., Herbert C., Maury J., Jacquet C., Balsse R., Esquerré-Tugayé M.T. (2001). Molecular characterization of *CLPT1*, a SEC4-like Rab/GTPase of the phytopathogenic fungus *Colletotrichum lindemuthianum* which is regulated by the carbon source. Gene.

[14] Zheng H., Chen S., Chen X., Liu S., Dang X., Yang C., Giraldo M.C., Oliveira-Garcia E., Zhou J., Wang Z. (2016). The small GTPase MoSec4 is involved in vegetative development and pathogenicity by regulating the extracellular protein secretion in *Magnaporthe oryzae*. Front. Plant Sci..

[15] Soldati T., Shapiro A.D., Svejstrup A.B., Pfeffer S.R. (1994). Membrane targeting of the small GTPase Rab9 is accompanied by nucleotide exchange. Nature.

[16] Ullrich O., Horiuchi H., Bucci C., Zerial M. (1994). Membrane association of Rab5 mediated by GDP-dissociation inhibitor and accompanied by GDP/GTP exchange. Nature.

[17] Segev N. (2001). Ypt/rab gtpases: Regulators of protein trafficking. Sci. STKE Signal Transduct. Knowl. Environ..

[18] Cherfils J., Zeghouf M. (2013). Regulation of small GTPases by GEFs, GAPs, and GDIs. Physiol. Rev..

[19] Barr F., Lambright D.G. (2010). Rab GEFs and GAPs. Curr. Opin. Cell Biol..

[20] Walch-Solimena C., Collins R.N., Novick P.J. (1997). Sec2p mediates nucleotide exchange on Sec4p and is involved in polarized delivery of post-Golgi vesicles. J. Cell Biol..

[21] Ortiz D., Medkova M., Walch-Solimena C., Novick P. (2002). Ypt32 recruits the Sec4p guanine nucleotide exchange factor, Sec2p, to secretory vesicles; evidence for a Rab cascade in yeast. J. Cell Biol..

[22] Sato Y., Shirakawa R., Horiuchi H., Dohmae N., Fukai S., Nureki O. (2007). Asymmetric coiled-coil structure with Guanine nucleotide exchange activity. Structure.

[23] Sato Y., Fukai S., Ishitani R., Nureki O. (2007). Crystal structure of the Sec4p. Sec2p complex in the nucleotide exchanging intermediate state. Proc. Natl. Acad. Sci. USA.

[24] Du L.L., Collins R.N., Novick P.J. (1998). Identification of a Sec4p GTPase-activating protein (GAP) as a novel member of a Rab GAP family. J. Biol. Chem..

[25] Chesneau L., Dupré S., Burdina A., Roger J., Le Panse S., Jacquet M., Cuif M.H. (2004). Gyp5p and Gyl1p are involved in the control of polarized exocytosis in budding yeast. J. Cell Sci..

[26] Gao X.D., Albert S., Tcheperegine S.E., Burd C.G., Gallwitz D., Bi E. (2003). The GAP activity of Msb3p and Msb4p for the Rab GTPase Sec4p is required for efficient exocytosis and actin organization. J. Cell Biol..

[27] Richardson P.M., Zon L.I. (1995). Molecular cloning of a cDNA with a novel domain present in the tre-2 oncogene and the yeast cell cycle regulators BUB2 and cdc16. Oncogene.

[28] Yan H., Huang J., Zhang H., Shim W.B. (2020). A Rab GTPase protein FvSec4 is necessary for fumonisin B1 biosynthesis and virulence in *Fusarium verticillioides*. Curr. Genet..

[29] Zheng H., Li L., Miao P., Wu C., Chen X., Yuan M., Fang T., Norvienyeku J., Li G., Zheng W. (2018). FgSec2A, a guanine nucleotide exchange factor of FgRab8, is important for polarized growth, pathogenicity and deoxynivalenol production in *Fusarium graminearum*. Environ. Microbiol..

[30] Zheng H., Li L., Yu Z., Yuan Y., Zheng Q., Xie Q., Li G., Abubakar Y.S., Zhou J., Wang Z. (2021). FgSpa2 recruits FgMsb3, a Rab8 GAP, to the polarisome to regulate polarized trafficking, growth and pathogenicity in *Fusarium graminearum*. New Phytol..

[31] Kwon M.J., Arentshorst M., Fiedler M., de Groen F.L.M., Punt P.J., Meyer V., Ram A.F.J. (2014). Molecular genetic analysis of vesicular transport in *Aspergillus niger* reveals partial conservation of the molecular mechanism of exocytosis in fungi. Microbiology.

[32] Callejas-Negrete O.A., Castro-Longoria E. (2019). The role of GYP-3 in cellular morphogenesis of *Neurospora crassa*: Analyzing its relationship with the polarisome. Fungal Genet. Biol. FGB.

[33] Lozowicka B., Iwaniuk P., Konecki R., Kaczynski P., Kuldybayev N., Dutbayev Y. (2022). Impact of diversified chemical and biostimulator protection on yield, health status, mycotoxin level, and economic profitability in spring wheat (*Triticum aestivum* L.) cultivation. Agronomy.

[34] Ordonez N., Seidl M.F., Waalwijk C., Drenth A., Kilian A., Thomma B.P., Ploetz R.C., Kema G.H. (2015). Worse comes to worst: Bananas and Panama disease--when plant and pathogen clones meet. PLoS Pathog..

[35] Hwang S.C., Ko W.H. (2004). Cavendish banana cultivars resistant to Fusarium wilt acquired through somaclonal variation in Taiwan. Plant Dis..

[36] Maryani N., Lombard L., Poerba Y.S., Subandiyah S., Crous P.W., Kema G.H.J. (2019). Phylogeny and genetic diversity of the banana *Fusarium* wilt pathogen *Fusarium oxysporum* f. sp. *cubense* in the Indonesian centre of origin. Stud. Mycol..

[37] Widinugraheni S., Niño-Sánchez J., van der Does H.C., van Dam P., García-Bastidas F.A., Subandiyah S., Meijer H.J.G., Kistler H.C., Kema G.H.J. (2018). A *SIX1* homolog in *Fusarium oxysporum* f. sp. *cubense* tropical race 4 contributes to virulence towards Cavendish banana. PLoS ONE.

[38] An B., Hou X., Guo Y., Zhao S., Luo H., He C., Wang Q. (2019). The effector *SIX8* is required for virulence of *Fusarium oxysporum* f.sp. *cubense* tropical race 4 to Cavendish banana. Fungal Biol..

[39] Yang S., Zhou X., Guo P., Lin Y., Fan Q., Zuriegat Q., Lu S., Yang J., Yu W., Liu H. (2021). The exocyst regulates hydrolytic enzyme secretion at hyphal tips and septa in the banana *Fusarium* Wilt Fungus *Fusarium odoratissimum*. Appl. Environ. Microbiol..

[40] Cheng C., Liu F., Sun X., Tian N., Mensah R.A., Li D., Lai Z. (2019). Identification of *Fusarium oxysporum* f. sp. *cubense* tropical race 4 (Foc TR4) responsive miRNAs in banana root. Sci. Rep..

[41] Yun Y., Liu Z., Yin Y., Jiang J., Chen Y., Xu J.R., Ma Z. (2015). Functional analysis of the *Fusarium graminearum* phosphatome. New Phytol..

[42] Leslie J.F., Summerell B.A. (2006). *Fusarium* laboratory workshops-A recent history. Mycotoxin Res..

[43] Dai Y., Cao Z., Huang L., Liu S., Shen Z., Wang Y., Wang H., Zhang H., Li D., Song F. (2016). CCR4-Not complex subunit Not2 plays critical roles in vegetative growth, conidiation and virulence in watermelon *Fusarium* wilt pathogen *Fusarium oxysporum* f. sp. *niveum*. Front. Microbiol..

[44] Yu J.H., Hamari Z., Han K.H., Seo J.A., Reyes-Domínguez Y., Scazzocchio C. (2004). Double-joint PCR: A PCR-based molecular tool for gene manipulations in filamentous fungi. Fungal Genet. Biol. FGB.

[45] Zheng W., Zheng H., Zhao X., Zhang Y., Xie Q., Lin X., Chen A., Yu W., Lu G., Shim W.B. (2016). Retrograde trafficking from the endosome to the trans-Golgi network mediated by the retromer is required for fungal development and pathogenicity in *Fusarium graminearum*. New Phytol..

[46] Zhao S., An B., Guo Y., Hou X., Luo H., He C., Wang Q. (2020). Label free proteomics and systematic analysis of secretome reveals effector candidates regulated by *SGE1* and *FTF1* in the plant pathogen *Fusarium oxysporum* f. sp. *cubense* tropical race 4. BMC Genom..

[47] Yun Y., Zhou X., Yang S., Wen Y., You H., Zheng Y., Norvienyeku J., Shim W.B., Wang Z. (2019). *Fusarium oxysporum* f. sp. *lycopersici* C(2)H(2) transcription factor FolCzf1 is required for conidiation, fusaric acid production, and early host infection. Curr. Genet..

[48] Eveleigh D.E., Mandels M., Andreotti R., Roche C. (2009). Measurement of saccharifying cellulase. Biotechnol. Biofuels.

[49] Chi M.H., Park S.Y., Kim S., Lee Y.H. (2009). A novel pathogenicity gene is required in the rice blast fungus to suppress the basal defenses of the host. PLoS Pathog..

[50] Fischer-Parton S., Parton R.M., Hickey P.C., Dijksterhuis J., Atkinson H.A., Read N.D. (2000). Confocal microscopy of FM4-64 as a tool for analysing endocytosis and vesicle trafficking in living fungal hyphae. J. Microsc..

[51] Riquelme M., Sánchez-León E. (2014). The Spitzenkörper: A choreographer of fungal growth and morphogenesis. Curr. Opin. Microbiol..

[52] Dong X., Ling N., Wang M., Shen Q., Guo S. (2012). Fusaric acid is a crucial factor in the disturbance of leaf water imbalance in Fusarium-infected banana plants. Plant Physiol. Biochem. PPB.

[53] Riquelme M. (2013). Tip growth in filamentous fungi: A road trip to the apex. Annu. Rev. Microbiol..

[54] Song W., Dou X., Qi Z., Wang Q., Zhang X., Zhang H., Guo M., Dong S., Zhang Z., Wang P. (2010). R-SNARE homolog MoSec22 is required for conidiogenesis, cell wall integrity, and pathogenesis of *Magnaporthe oryzae*. PLoS ONE.

[55] Bickle M., Delley P.A., Schmidt A., Hall M.N. (1998). Cell wall integrity modulates *RHO1* activity via the exchange factor ROM2. EMBO J..

[56] Lussier M., White A.M., Sheraton J., di Paolo T., Treadwell J., Southard S.B., Horenstein C.I., Chen-Weiner J., Ram A.F., Kapteyn J.C. (1997). Large scale identification of genes involved in cell surface biosynthesis and architecture in *Saccharomyces cerevisiae*. Genetics.

[57] Ram A.F., Wolters A., Ten Hoopen R., Klis F.M. (1994). A new approach for isolating cell wall mutants in *Saccharomyces cerevisiae* by screening for hypersensitivity to calcofluor white. Yeast.

[58] Zheng H., Zheng W., Wu C., Yang J., Xi Y., Xie Q., Zhao X., Deng X., Lu G., Li G. (2015). Rab GTPases are essential for membrane trafficking-dependent growth and pathogenicity in *Fusarium graminearum*. Environ. Microbiol..

[59] Li C., Yang J., Li W., Sun J., Peng M. (2017). Direct root penetration and rhizome vascular colonization by *Fusarium oxysporum* f. sp. *cubense* are the key steps in the successful infection of Brazil Cavendish. Plant Dis..

[60] Nair J., Müller H., Peterson M., Novick P. (1990). Sec2 protein contains a coiled-coil domain essential for vesicular transport and a dispensable carboxy terminal domain. J. Cell Biol..

[61] De Antoni A., Schmitzová J., Trepte H.H., Gallwitz D., Albert S. (2002). Significance of GTP hydrolysis in Ypt1p-regulated endoplasmic reticulum to Golgi transport revealed by the analysis of two novel Ypt1-GAPs. J. Biol. Chem..

