# Supplementary material for: Small GTPase FoSec4-Mediated Protein Secretion Is Important for Polarized Growth, Reproduction and Pathogenicity in the Banana Fusarium Wilt Fungus Fusarium odoratissimum
Source: J Fungi (Basel). 2022 Aug 20;8(8):880. doi: 10.3390/jof8080880 (PMC9410047; doi:10.3390/jof8080880)
Supplement: Supplementary file 1 [file jof-08-00880-s001.zip › jof-1811120-supplementary.pdf]

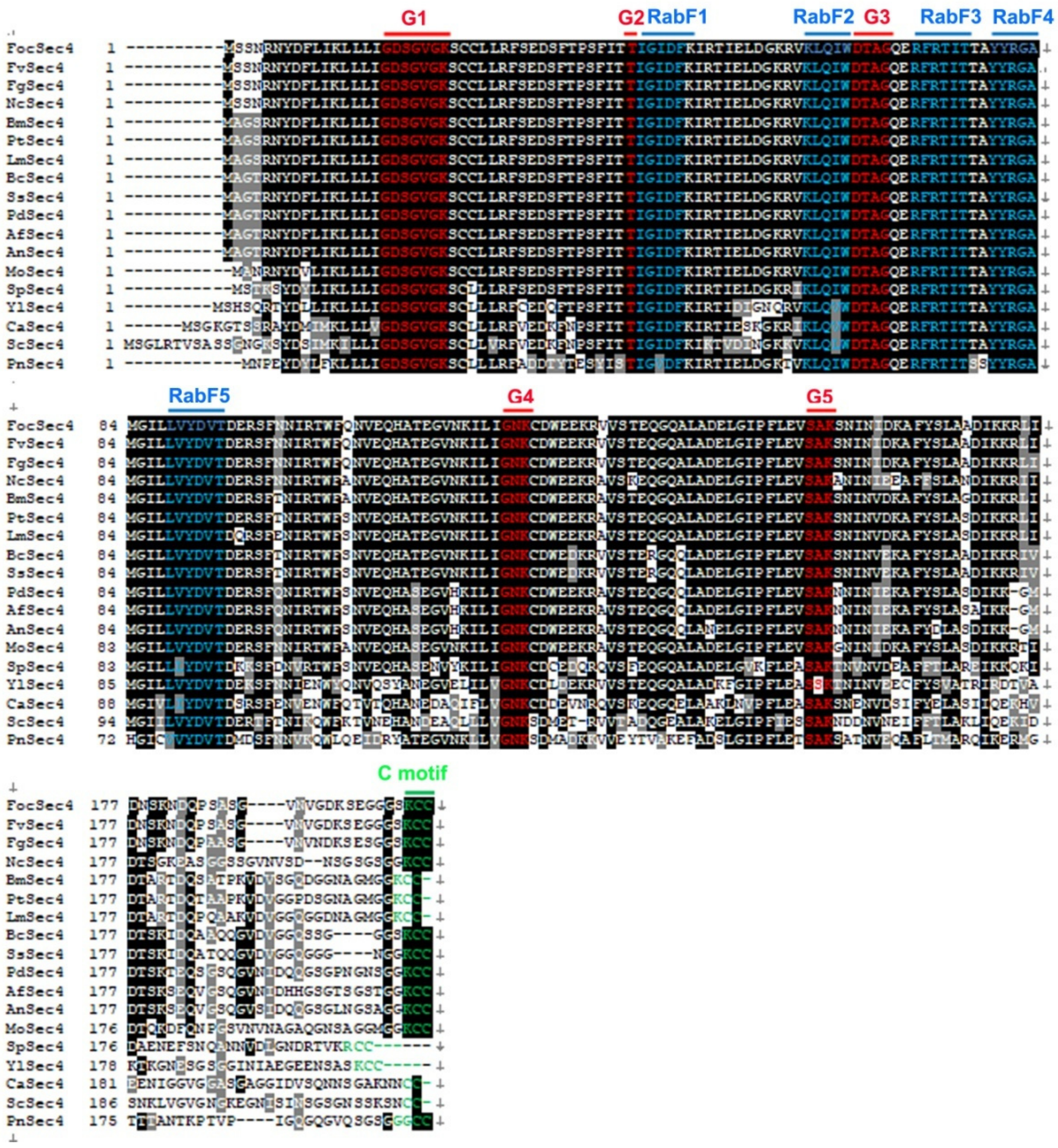

Figure S1 Multiple alignment of Sec4 homologs in some fungi species. The fungi species consists of *Fusarium odoratissimum* (Fo), *Fusarium verticillioides* (Fv), *Fusarium graminearum* (Fg), *Neurospora crassa* (Nc), *Magnaporthe oryzae* (Mo), *Aspergillus niger* (An), *Aspergillus fumigates* (Af), *Penicillium digitatum*(Pd), *Sclerotinia sclerotiorum* (Ss), *Botrytis cinerea* (Bc), *Bipolaris maydis* (Bm), *Leptosphaeria maculans* (Lm), *Pyrenophora tritici-repentis* (Pt), *Schizosaccharomyces pombe* (Sp), *Parastagonospora noaorum* (Pn), *Saccharomyces cereviseiae* (SC), *Candida albicans* (Ca), *Yarrowia lipolytica* (Yl). Five conserved domains labeled as G1 to G5 were in red, five Rab-specific labeled as RabF1 to RabF5 were in blue and a C-terminal domain labeled as C motif was in green.

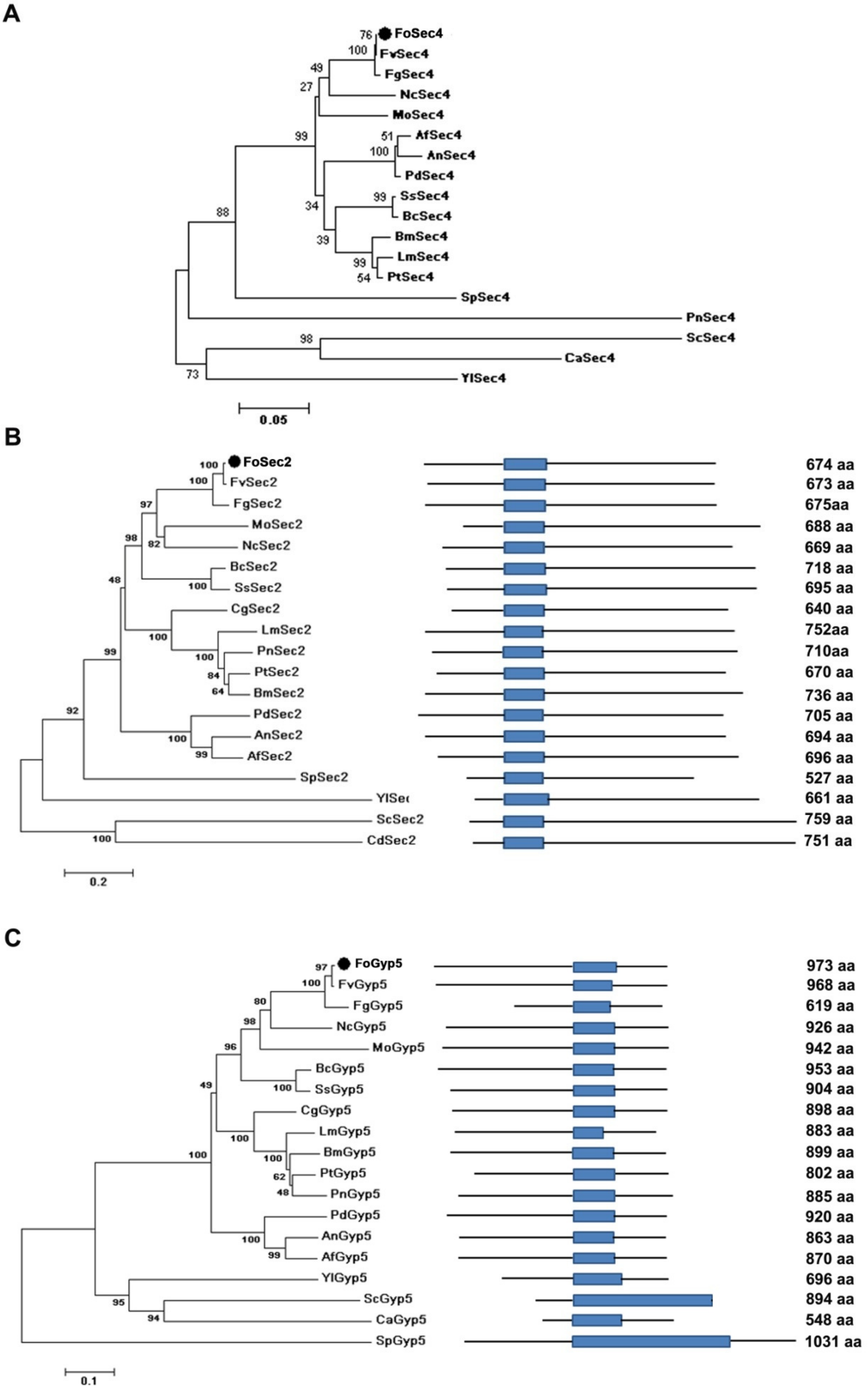

Figure S2 Phylogenetic analysis of Sec4, Sec2 and Gyp5 homologs in some fungi species. The fungi species consists of *Fusarium odoratissimum* (Fo), *Fusarium verticillioides*

(Fv), *Fusarium graminearum* (Fg), *Neurospora crassa* (Nc), *Magnaporthe oryzae* (Mo), *Aspergillus niger* (An), *Aspergillus fumigates* (Af), *Penicillium digitatum*(Pd), *Sclerotinia sclerotiorum* (Ss), *Botrytis cinerea* (Bc), *Bipolaris maydis* (Bm), *Leptosphaeria maculans* (Lm), *Pyrenophora tritici-repentis* (Pt), *Schizosaccharomyces pombe* (Sp), *Parastagonospora noaorum* (Pn), *Saccharomyces cereviseiae* (SC), *Candida albicans* (Ca), *Yarrowia lipolytica* (Yl). Evolutionary analyses were conducted in MEGA7.0. The evolutionary history was inferred based on the amino acid sequences by using a neighbor-joining method. The numbers at nodes showed the percentage of their occurrence in 1,000 bootstrap replicates. **(A)** Phylogenetic analysis of Sec4. **(B)** Phylogenetic analysis of Sec2 homologs (left panel). Values on the branches of clusters represent the results of bootstrap analysis. Schematic representations of the structures of Sec2 proteins (right panel). The Sec2 domain is shown in blue. **(C)** Phylogenetic analysis of Gyp5 homologs (left panel). Values on the branches of clusters represent the results of bootstrap analysis. Schematic representations of the structures of Gyp5 proteins (right panel). The TBC domain is shown in blue.

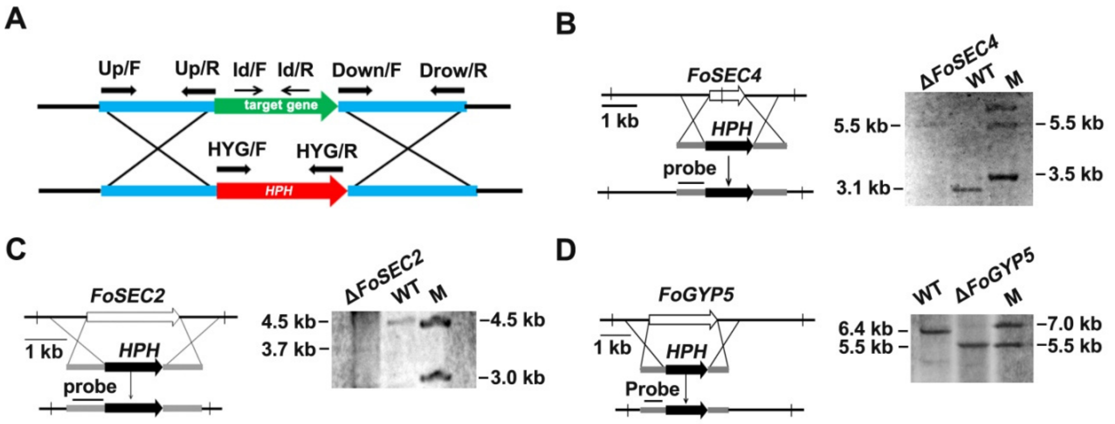

Figure S3 Southern blot analyses of targeted gene deletion mutants. (A) The split-marker approach was used to delete the targeted genes. (B) Targeted gene deletion of FoSec4, *Pst* I and *Kpn* I digested DNAs showed a 3.1 kb in the WT and a 5.5 kb band in mutants. (C) Targeted gene deletion of FoSec2, *Dra* I digested DNAs showed a 4.5 kb in the WT and a 3.7 kb band in mutants. (D) Targeted gene deletion of FoGyp5, *Dra* I and *Kas* I digested DNAs showed a 6.4 kb in the WT and a 5.5 kb band in mutants.

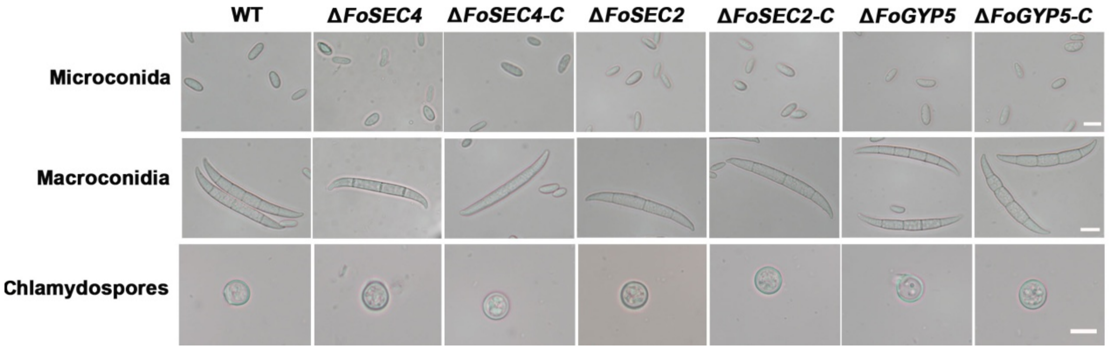

Figure S4 The morphology of the microconidia, macroconidia and chlamydospores in the wild-type(WT), ΔFoSEC4, ΔFoSEC2, ΔFoGYP5, ΔFoSEC4-C, ΔFoSEC2-C and ΔFoGYP5-C strains. Bar=10 μm.

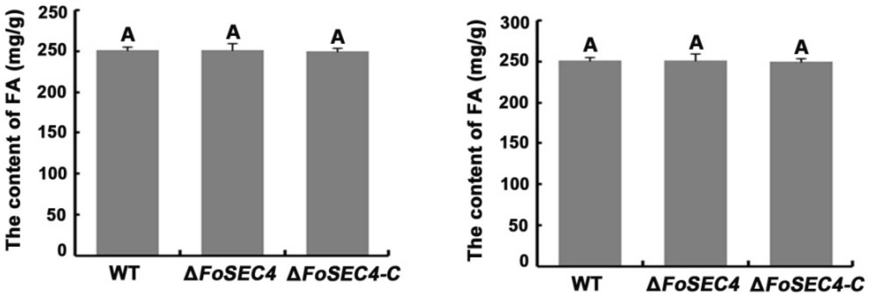

Figure S5 Fusaric acid production levels by wild-type (WT), Δ*FoSEC4* and Δ*FoSEC4-C* strains were analyzed by HPLC. Fusaric acid levels (mg g^{-1}) were measured per mycelial dry weight.

Table S1 strains used in the study.

**Strain**	**Genotype**	**Source**
WT	*Fusarium odoratissimum* wild type strain	This study
ΔFoSEC4	*FoSEC4* deletion mutant of *F. odoratissimum*	This study
WT-GFP	The wild-type stain labeled with GFP	This study
ΔFoSEC4-GFP	The *FoSEC4* deletion mutant labeled with GFP	This study
ΔFoSEC2	*FoSEC2* deletion mutant of *F. odoratissimum*	This study
ΔFoGYP5	*FoGYP5* deletion mutant of *F. odoratissimum*	This study
ΔFoSEC4-C	Complemented strain with *FoSEC4*	This study
ΔFoSEC2-C	Complemented strain with *FoSEC2*	This study
ΔFoGYP5-C	Complemented strain with *FoGYP5*	This study
ΔFoSEC2-1	Complemented strain with FoSec2 without the Sec2 domain	This study
ΔFoSEC2-2	Complemented strain with amino acids 184-677 of FoSec2	This study
ΔFoSEC2-3	Complemented strain with amino acids 1-277 of FoSec2	This study
ΔFoGYP5-1	Complemented strain with FoGyp5 without the TBC domain	This study
ΔFoGYP5-2	Complemented strain with amino acids 580-973 of FoGyp5	This study
ΔFoGYP5-3	Complemented strain with amino acids 184-677 of FoGyp5	This study

Table S2 Primers used in this study

Name	Sequence(5'→3')	Relevant characteristics
FoSEC4-up	F:GAACCCGAGTTTGCAGAAAA<br>R:CATTCATTGTTGACCTCCACTAGCTCCACCAAGAGTGAGAGTAGCGGAA	PCR primers to amplify *FoSEC4* upstream fragment for the construction of *FoSEC4* deletion mutant
FoSEC4-down	F:GCAAAGGAATAGAGTAGATGCCGACCGCCGAATACCCTAAGGAGTTCG<br>R:AAGGCGATCATAAAGCAGGA	PCR primers to amplify *FoSEC4* downstream fragment for the construction of *FoSEC4* deletion mutant
FoSEC4-nest	F:TCAAAGATAGGCGCCACAAT<br>R:AAGTGGCATGGCGATTCATT	PCR primers to amplify upstream-HPH-downstream fragment for deletion of *FoSEC4*
FoSEC4-id	F:ATTATTCACTGCATGCGACC<br>R:ATGTTGCTCTTGGCGGAGA	PCR primers for identification *FoSEC4* deletion transformants
FoGYP5-up	F:CCCATCAAATGCCCGAGAAG<br>R:CATTCATTGTTGACCTCCACTAGCTCCAAGACGGAAGAGAGATGCTGT	PCR primers to amplify *FoGYP5* upstream fragment for the construction of *FoGYP5* deletion mutant
FoGYP5-down	F:GCAAAGGAATAGAGTAGATGCCGACCGGAAAATCGAAAAAACTCCCG<br>R:TGCCTACTACCGTGCGTAAT	PCR primers to amplify *FoGYP5* downstream fragment for the construction of *FoGYP5*deletion mutant
FoGYP5-nest	F:TGAAGCACGAAGACAAGCAC<br>R:GACTCTACGCCGTTGTTTCC	PCR primers to amplify upstream-HPH-downstream fragment for deletion of *FoGYP5*
FoGYP5-id	F: GATGGAGACTCACTACCCGG<br>R: AGATCCCGGCGTATTGTCTT	PCR primers for identification *FoGYP5* deletion transformants
HYG	F:TGGAGCTAGTGGAGGTCAACAATGAATG<br>R:CGGTCGGCATCTACTCTATTCCTTTGC	PCR primers for amplification of hygromycin resistance gene (*HPH*)
FoSEC2-up	F:CGAGTGAGGCGAAAAGGAAG<br>R:CATTCATTGTTGACCTCCACTAGCTCCAGGGCGTATGTGTGTCAGTTG	PCR primers to amplify *FoSEC2* upstream fragment for the construction of *FoSEC2* deletion mutant
FoSEC2-down	F:GCAAAGGAATAGAGTAGATGCCGACCGGTGATATCGACGCCACTGTG<br>R:AAGGCTGGAAAGGAGAAAGC	PCR primers to amplify *FoSEC2* downstream fragment for the construction of *FoSEC2* deletion mutant
FoSEC2-nest	F:TGCCTTTCAAGTCCCTGGAT<br>R:AAGGAGGGGAGACGAGGTTA	PCR primers to amplify upstream-HPH-downstream fragment for deletion of *FoSEC2*

FoSEC2-id	F:ACGTCACCAGCCAACATTTC<br>R:GCTTGCACCTTCTTCTCTCG	PCR primers for identification *FoSEC2* deletion transformants
FoSEC4CA-up	F:GAACCCGAGTTTGCAGAAAA<br>R:ACCGGCGGTATCCCAGATCTGC	PCR primers to amplify upstream fragment for the construction of *FoSEC4CA* point mutant
FoSEC4CA-down	F:GCAGATCTGGGATACCGCCGGTCTGGAGCGTTTCCGCACCAT<br>R:TCCTAGCCATGTGCTACTTGA	PCR primers to amplify downstream fragment for the construction of *FoSEC4CA* point mutant
FoSEC4DN-up	F:GAACCCGAGTTTGCAGAAAA<br>R:GCCAATTAGAATCTTGTTGACA	PCR primers to amplify upstream fragment for the construction of *FoSEC4DN* point mutant
FoSEC4DN-down	F:TGTCAACAAGATTCTAATTGGCATCAAGTGCGACTGGGAGGA<br>R:AGAGCAAACGGGTCAGAAAG	PCR primers to amplify downstream fragment for the construction of *FoSEC4DN* point mutant
G418-1	F:TCTAGATTAACGCTTACAATTTCCA<br>R:GCCCAATAGCAGCCAGTCC	PCR primers for amplification upstream of neomycin resistance gene
G418-2	F:CAACAACACGCATCATCCCA<br>R:TCAGAAGAACTCGTCAAGAA	PCR primers for amplification downstream of neomycin resistance gene
FoSEC4-NP	F:GAACCCGAGTTTGCAGAAAA<br>R:TCCTCGCCCTTGCTCACCATGTTGACCAAGAGTGAGAGTAGCG	PCR primers to amplify *FoSEC4* upstream fragment for the construction of *GFP-FoSec4*
FoSEC4-GFP	F:GGCGGAGGCGGCGGAGGCGGAGGCGGAGGCTCGAGTAATCGTAACTATG<br>R:CGAACTCCTTAGGGTATTCGG	PCR primers to amplify *FoSEC4* downstream fragment for the construction of *GFP-FoSec4*
GFP-1	F:ATGGTGAGCAAGGGCGAGGA<br>R:ATGGGGGTGTTCTGCTGGTA	PCR primers for amplification upstream of GFP
GFP-2	F:AAGCAGCACGACTTCTTCAA<br>R:GCCTCCGCCTCCGCCTCCGCCGCCTCCGCCCTTGTACAGCTCGTCCATGC	PCR primers for amplification downstream of GFP
FoSEC2-NP	F:CGAGTGAGGCGAAAAGGAAG<br>R:TCCTCGCCCTTGCTCACCATTGTGATCGGGCGTATGTGTG	PCR primers to amplify *FoSEC4* upstream fragment for the construction of *GFP-FoSec2*
FoSEC2-GFP	F:GGCGGAGGCGGCGGAGGCGGAGGCGGAGGCGACTCGTACGTTGCCATCAT<br>R:ACGTCACCAGCCAACATTTC	PCR primers to amplify *FoSEC4* downstream fragment for the construction of *GFP-FoSec2*

FoGyp5-GFP	F:AGGGAACAAAAGCTGGGTACCAGCCAGAAAAGGACTCGTGA<br>R:GCCCTTGCTCACCATAAGCTTTGCAAACTGACGCTTCAAGT	PCR primers to amplify *FoGYP5* full fragment for the construction of *FoGyp5-GFP*
BD-SEC4	F:TCAGAGGAGGACCTGCATATGATGTCGAGTAATCGTAACT<br>R:TCGACGGATCCCCGGGAATTCTTAGCAGCACTTGCTGCCG	PCR primers for BD-Sec4 constructs using pGBKT7
AD-GYP5(N)	F:GTACCAGATTACGCTCATATGATGTCGCAACCGCAAGATG<br>R:ATGCCCACCCGGGTGGAATTCTTATAGTGTCGCCGCGTCCT	PCR primers to amplify N teminal of FoGyp5 for AD-GYP5 N teminal constructs
AD-GYP5(TBC)	F:GTACCAGATTACGCTCATATGATGCAGAAACTGGAAAAGAC<br>R:ATGCCCACCCGGGTGGAATTCTTACAGGAGAATGCTGGCAT	PCR primers to amplify TBC domain of FoGyp5 for AD-GYP5 TBC domain constructs
AD-GYP5(C)	F:GTACCAGATTACGCTCATATGATGGGATTATCTGACATGCAG<br>R:ATGCCCACCCGGGTGGAATTCTCATGCAAACTGACGCTTC	PCR primers to amplify C teminal of FoGyp5 for AD-GYP5 C teminal constructs
AD-SEC2(N)	F:GTACCAGATTACGCTCATATGATGGACTCATTTATTCCAA<br>R:ATGCCCACCCGGGTGGAATTCTTAGGCCTGCCACACCTTCTT	PCR primers to amplify N teminal of FoSec2 for AD-SEC2 N teminal constructs
AD-SEC2(Sec2)	F:GTACCAGATTACGCTCATATGATGAAGGTCGCCGCGGAACGTCAAA<br>R:ATGCCCACCCGGGTGGAATTCTTAATGATCTCCTCGTTCCGAAG	PCR primers to amplify Sec2 domain of FoSec2 for AD-SEC2 Sec2 domain constructs
AD-SEC2(C)	F:GTACCAGATTACGCTCATATGATGAATAACCCCACTGCTCCCTCT<br>R:ATGCCCACCCGGGTGGAATTCTTAATCCGTAGTCGGAATC	PCR primers to amplify C teminal of FoSec2 for AD-SEC2 C teminal constructs
AD-SEC2(N-Sec2)	F:GTACCAGATTACGCTCATATGATGGACTCATTTATTCCAA<br>R:ATGCCCACCCGGGTGGAATTCTTAATGATCTCCTCGTTCCGAAG	PCR primers to amplify N-Sec2 region of FoSec2 for AD-SEC2 N-Sec2 region constructs
AD-SEC2(Sec2-C)	F:GTACCAGATTACGCTCATATGATGAAGGTCGCCGCGGAACGTCAAA	PCR primers to amplify Sec2-C region of FoSec2 for AD-SEC2 Sec2-C region constructs

	R:ATGCCCACCCGGGTGGAATTCTTA ATCCGTAGTCGGAATC	
AD- EXO70	F:GTACCAGATTACGCTCATATGATGT CGGTCGGACTCTCTG	PCR primers for AD- EXO70 constructs
	R:ATGCCCACCCGGGTGGAATTCTCA GGAAGCCAAACTGGAA	
AD- GYP1p	F:GTACCAGATTACGCTCATATGATGG TCCAAGTCGACCGAT	PCR primers for AD- GYP1p constructs
	R:ATGCCCACCCGGGTGGAATTCTCA CAGTTGTAGGTTCGTA	
AD- GYP5	F:GTACCAGATTACGCTCATATGATGT CGCAACCGCAAGATG	PCR primers for AD- GYP5 constructs
	R:ATGCCCACCCGGGTGGAATTCTCA TGCAAACTGACGCTTC	
AD- KES1	F:GTACCAGATTACGCTCATATGATGT CCTCTTCTACAGCCC	PCR primers for AD- KES1 constructs
	R:ATGCCCACCCGGGTGGAATTCCTAT CGACCAAGAAGCTCC	
AD- MRS6	F:GTACCAGATTACGCTCATATGATGG AATCCCTCTCAGACA	PCR primers for AD- MRS6 constructs
	R:ATGCCCACCCGGGTGGAATTCCTA GTCGAAGGGATCATCA	
AD- MSB3	F:GTACCAGATTACGCTCATATGATGA CTCCCCTCGCTATTT	PCR primers for AD- MSB3 constructs
	R:ATGCCCACCCGGGTGGAATTCCTAT CCCACGTTCCACGTC	
AD- MYO2	F: GTACCAGATTACGCTCATATGATGGC ATCGGCCTACGAGG	PCR primers for AD- MYO2 constructs
	R:ATGCCCACCCGGGTGGAATTCCTA CTGCTCTACTTCCACC	
AD-SEC2	F:GTACCAGATTACGCTCATATGATGG ACTCATTTATTCCAA	PCR primers for AD- SEC2 constructs
	R:ATGCCCACCCGGGTGGAATTCTTA ATCCGTAGTCGGAATC	
AD- SEC3	F:GTACCAGATTACGCTCATATGATGG ACCGCCCAAACGG	PCR primers for AD- SEC3 constructs
	R:ATGCCCACCCGGGTGGAATTCTCA	

	ACGGAATGCGGCCTTG	
AD- SEC15	F:GTACCAGATTACGCTCATATGATGCCGCGACGTCCGCCTG<br>R:ATGCCCACCCGGGTGGAATTCTCATGTTCGGTTTATGCCA	PCR primers for AD- SEC15 constructs
AD-SRO7p	F:GTACCAGATTACGCTCATATGATGGCGGCTTTCCTGCGCG<br>R:ATGCCCACCCGGGTGGAATTCTTAAAAGAACTTGCTCTTA	PCR primers for AD- SRO7p constructs
AD- YPT1	F:GTACCAGATTACGCTCATATGATGAACCCTGAATACGACT<br>R:ATGCCCACCCGGGTGGAATTCTTAGCAGCAGCTGTTGTTG	PCR primers for AD- YPT1 constructs
FoGyp5-1-up	F:AGGGAACAAAAGCTGGGTACCAGCCAGAAAAGGACTCGTGA<br>R:TAGTGTCGCCGCGTCCTCCTTGTCCTTCCGCTTCCGCTCA	PCR primers to amplify upstream fragment of FoGyp5 without the TBC domain for the construction of Δ*FoGYP5-1*
FoGyp5-1-down	F:AGGAGGACGCGGCGACACTAGGATTATCTGACATGCAGCA<br>R:GCCCTTGCTCACCATAAGCTTTGCAAACTGACGCTTCAAGT	PCR primers to amplify downstream fragment of FoGyp5 without the TBC domain for the construction of Δ*FoGYP5-1*
FoGyp5-2-up	F:AGGGAACAAAAGCTGGGTACCAGCCAGAAAAGGACTCGTGA<br>R:GTCTTTTCCAGTTTCTGCATGGTTGAGGAGGAGGGTGTTG	PCR primers to amplify upstream fragment of FoGyp5 without N teminal for the construction of Δ*FoGYP5-2*
FoGyp5-2-down	F:ATGCAGAAACTGGAAAAGACAATACGCCGGGATCTTGGTG<br>R:GCCCTTGCTCACCATAAGCTTTGCAAACTGACGCTTCAAGT	PCR primers to amplify downstream fragment of FoGyp5 without N teminal for the construction of Δ*FoGYP5-2*
FoGyp5-3	F:AGGGAACAAAAGCTGGGTACCAGCCAGAAAAGGACTCGTGA<br>R:GCCCTTGCTCACCATAAGCTTCAGGAGAATGCTGGCATTCT	PCR primers to amplify FoGyp5 without C teminal for the construction of Δ*FoGYP5-3*
FoSec2-1-up	F:AGGGAACAAAAGCTGGGTACCAAACTCGGCCTAGCTTCTGT<br>R:GGCCTGCCACACCTTCTTTTCGTCCTCAACGGTGTGCTTG	PCR primers to amplify upstream fragment of FoSec2 without the Sec2 domain for the construction of Δ*FoSEC2-1*

FoSec2-1-down	F:AAGAAGGTGTGGCAGGCCAATAACCCCACTGCTCCCTC<br>R:GCCCTTGCTCACCATAAGCTTATCCGTAGTCGGAATCGTGA	PCR primers to amplify downstream fragment of FoSec2 without the TBC domain for the construction of Δ*FoSEC2-1*
FoSec2-2-up	F:AGGGAACAAAAGCTGGGTACCAAACTCGGCCTAGCTTCTGT<br>R:CGTTCCGCGGCGACCTTCATTGTGATCGGGCGTATGTGTG	PCR primers to amplify upstream fragment of FoSec2 without N-teminal for the construction of Δ*FoSEC2-2*
FoSec2-2-down	F:ATGAAGGTCGCCGCGGAACGTCAAAAGCGACTTGACACAG<br>R: R:GCCCTTGCTCACCATAAGCTTATCCGTAGTCGGAATCGTGA	PCR primers to amplify downstream fragment of FoSec2 without N-teminal for the construction of Δ*FoSEC2-2*
FoSec2-3	F:AGGGAACAAAAGCTGGGTACCAAACTCGGCCTAGCTTCTGT<br>R:GCCCTTGCTCACCATAAGCTTATGATCTCCTCGTTCCGAAG	PCR primers to amplify FoSec2 without C-teminal for the construction of Δ*FoSEC2-3*
